# Sensitization of the Nociceptive System in Complex Regional Pain Syndrome

**DOI:** 10.1371/journal.pone.0154553

**Published:** 2016-05-05

**Authors:** Maren Reimer, Torge Rempe, Carolina Diedrichs, Ralf Baron, Janne Gierthmühlen

**Affiliations:** Division of Neurological Pain Research and Therapy, Department of Neurology, University Hospital of Schleswig-Holstein, Campus Kiel, Germany; University of Würzburg, GERMANY

## Abstract

**Background:**

Complex regional pain syndrome type I (CRPS-I) is characterized by sensory, motor and autonomic abnormalities without electrophysiological evidence of a nerve lesion.

**Objective:**

Aims were to investigate how sensory, autonomic and motor function change in the course of the disease.

**Methods:**

19 CRPS-I patients (17 with acute, 2 with chronic CRPS, mean duration of disease 5.7±8.3, range 1–33 months) were examined with questionnaires (LANSS, NPS, MPI, Quick DASH, multiple choice list of descriptors for sensory, motor, autonomic symptoms), motor and autonomic tests as well as quantitative sensory testing according to the German Research Network on Neuropathic Pain at two visits (baseline and 36±10.6, range 16–53 months later).

**Results:**

CRPS-I patients had an improvement of sudomotor and vasomotor function, but still a great impairment of sensory and motor function upon follow-up. Although pain and mechanical detection improved upon follow-up, thermal and mechanical pain sensitivity increased, including the contralateral side. Increase in mechanical pain sensitivity and loss of mechanical detection were associated with presence of ongoing pain.

**Conclusions:**

The results demonstrate that patients with CRPS-I show a sensitization of the nociceptive system in the course of the disease, for which ongoing pain seems to be the most important trigger. They further suggest that measured loss of function in CRPS-I is due to pain-induced hypoesthesia rather than a minimal nerve lesion. In conclusion, this article gives evidence for a pronociceptive pain modulation profile developing in the course of CRPS and thus helps to assess underlying mechanisms of CRPS that contribute to the maintenance of patients’ pain and disability.

## Introduction

Complex regional pain syndrome (CRPS) is characterized by sensory, motor and autonomic abnormalities [1, 2]. Two types can be distinguished: Type I without and type II with electrophysiological evidence of major nerve lesion [3]. There has been a discussion as to whether CRPS type I (CRPS-I) represents a neuropathic pain syndrome because it by definition presents without any major nerve lesion and does thus not fulfill the criteria for neuropathic pain [4]. Indeed, CRPS-I has been excluded as a neuropathic pain entity in the recently published guidelines on pharmacological treatment of neuropathic pain [5]. Nevertheless, in a recent cross-sectional study it has been shown that somatosensory profiles of patients with CRPS-I are similar to those with CRPS-II despite evidence of a major nerve lesion in CRPS-II [6]. Most of CRPS-I and II patients show a combination of gain and loss, i.e. increased sensitivity to thermal and mechanical stimuli, with pressure pain hyperalgesia being most frequent in combination with a loss of thermal and mechanical detection. The loss in around 63% of CRPS-I patients was suggested to be either due to a minimal nerve lesion or pain-induced hypoesthesia [6] resulting in similar pathophysiological mechanisms as assumed for CRPS-II. The diagnostic Budapest criteria differentiate between presence of signs and symptoms, i.e. symptoms are what the patient reports, signs are findings upon clinical examination [7].

To date the chronological sequence of somatosensory as well as motor and autonomic abnormalities with differentiation between examination of signs and symptoms in the course of the disease has been poorly studied. Additionally, a limitation of the few studies that evaluated the change of clinical abnormalities in the course of CRPS following treatment is that they used different diagnostic criteria and outcomes and mainly performed retrospective analyses of cases or used a cross-sectional study design [8].

Thus, the aims of this study were to investigate if and how somatosensory, autonomic and motor signs and symptoms change in the course of the disease. This might help to determine whether loss of detection in CRPS-I is due to a nerve lesion or pain-induced hypoesthesia. Within this study we could demonstrate that patients with CRPS-I show a sensitization of the nociceptive system in the course of the disease. Furthermore, this study's results suggest that measured loss of function in CRPS-I is due to pain-induced hypoesthesia rather than a minimal nerve lesion.

## Methods

### Patients

The study examined 19 patients with CRPS-I of the upper extremity. Recruitment consisted of all patients with CRPS type I of the upper extremity who had been included into the database of the German Research Network on Neuropathic Pain in Kiel, Germany between 2004 and 2007 [6] (n = 45) and who agreed to participate in a follow-up examination (n = 19). Inclusion was restricted to patients with upper limb CRPS to make the investigated patient sample as homogenous as possible for design and analysis of the study.

A diagnosis of CRPS-I and inclusion into the database was made when (A) a glove-like distal distribution of pain, signs and symptoms that spread beyond the innervation territory of a single nerve was present, (B) Budapest criteria for clinical diagnosis were fulfilled [7] and (C) no overt nerve lesion was detectable [3] upon electrophysiological examination.

Patients with any neurological comorbidity that could otherwise influence testing results such as polyneuropathy, diabetes, vascular disease etc. as well as patients with skin lesions or dermatological disorders in the areas to be tested or with difficulties in German language skills were excluded from the study.

The study was in accordance with the Declaration of Helsinki and approved by the Institutional Review Board of the Faculty of Medicine at Christian-Albrechts-University of Kiel. All patients gave written informed consent to take part in the study.

### Experimental set-up

The initial visit’s (visit 1) dataset was provided by the database of the German Research Network on Neuropathic Pain. The examination of both the initial visit and the follow-up visit was performed in a quiet room with a constant temperature of 21°C and included an anamnesis with a detailed assessment of subjective symptoms, a clinical examination with an assessment of objective signs, different questionnaires and quantitative-sensory testing (QST). Both examinations followed an identical algorithm with the exception of assessment of sleep disturbances, impairment of daily life and the ability to work as well as Quick-DASH (Disabilities of the Arm, Shoulder and Hand), long-term skin temperature measurement and finger tapping, which were not part of the database of the German Research Network on Neuropathic Pain and were therefore only assessed upon follow-up (**Table 1, S1 and S2 Protocols**). At the beginning of the follow-up visit, patients were asked for presence of any new medical issues that might have occurred since the first examination. Afterwards, all patients received the different questionnaires and anamnesis was taken including questions regarding course and treatment of the disease, status of current pain as well as presence of inflammatory, sensory, autonomic and motor abnormalities for evaluation of symptoms. Next, a clinical-neurological examination was performed to screen for new comorbidities that might have developed in the meantime and could interfere with testing results. Then, quantitative-sensory testing (QST) was executed [9]. At the end of the investigation, the patients were equipped with small loggers affixed to the small fingers of both hands for long-term skin temperature measurement.

**Table 1:** Investigations at visit 1 and 2 (follow-up).

	Visit 1	Visit 2 (follow-up)
**Medical history(symptoms)**		
Pain intensity	X	X
Pain characteristics	X	X
Sensory abnormalities	X	X
Motor abnormalities	X	X
Autonomic abnormalities	X	X
Sleep disturbances		X
Impairment of daily life		X
Ability to work		X
**Questionnaires**		
LANSS	X	X
NPS	X	X
MPI	X	X
Quick DASH		X
**Clinical signs**		
Inflammatory signs	X	X
QST	X	X
Autonomic abnormalities	X	X
Skin temperature	X	X
Long-term skin temperature		X
Motor abnormalities	X	X
Range of motion	X	X
Finger tapping		X
**CRPS severity Score**	X	X
**Budapest criteria**	X	X

LANSS: Leeds Assessment of Neuropathic Symptoms and Signs, NPS: Neuropathic Pain Scale, MPI: German Multidimensional Pain Inventory, Quick DASH: Assessment of Disabilities of the Arm, Shoulder and Hand, QST: Quantitative Sensory Testing, CRPS: Complex regional pain syndrome.

### Assessment of symptoms

Upon follow-up, subjects were asked (dichotomous yes/no questions) for presence of sleep disturbances and social retreat due to CRPS as well as their ability to work. Subjective estimation of disease improvement/aggravation was evaluated by the patients using a numerical rating scale (NRS) between -10 and + 10 representing worst aggravation and best improvement, respectively. Impairment of daily life due to CRPS was estimated on the NRS with 0 = no impairment and 10 = the maximum impairment imaginable. Mild impairment was defined NRS 0–3, moderate 4–6 and severe 7–10.

Although it is often questioned whether CRPS type I represents a neuropathic pain entity, clinical experience and our former research [6] suggest that CRPS types I and II share similar pathophysiological mechanisms. Therefore, two questionnaires (LANSS and NPS) were included to determine the contribution of neuropathic mechanisms to the patients’ pain.

#### Leeds Assessment of Neuropathic Symptoms and Signs (LANSS)

Upon first and second examination, patients completed the German versions of LANSS [10]. The LANSS contains 5 symptom items based on an interview of the patient and 2 clinical examination items [10]. It can be used as a screening tool to identify patients with pain of predominantly neuropathic origin [11],[12]. The LANSS has been tested and validated in several settings [13–15] with sensitivity and specificity ranging from 82% to 91% and 80% to 94%, respectively, compared to clinical diagnosis [11].

#### Neuropathic Pain Scale (NPS)

The NPS [16] was completed upon visit 1 and 2. It includes ten pain quality items rated on a 0–10 Likert scale and one temporal assessment of pain. In contrast to the LANSS, the NPS is a measurement rather than a screening tool, focusing on characteristic aspects and temporal assessment of neuropathic pain [16]. The NPS has been validated specifically for neuropathic pain [16–19].

#### Multidimensional Pain Inventory for assessment of neuropathic pain and psychological impairment (MPI)

The German version of the MPI [20, 21] was used upon visit 1 and 2. Three parts of the inventory, comprised of 12 scales, examine the impact of pain on the patients' lives, the responses of others to the patients' communications of pain, and the extent to which patients participate in common daily activities [20]. Scores were compared between visit 1 and 2.

#### Assessment of Disabilities of the Arm, Shoulder and Hand (Quick DASH)

Impairment of motor function was investigated with the Quick DASH [22]. For evaluation of Quick DASH, suggested analysis was used, i.e. the values for the different questions were added to a raw value and the final value calculated as follows: ((raw value/number of answered questions)-1) x 25 = Quick DASH value (0–100). As Quick DASH data was not part of the database of the German Research Network on Neuropathic Pain, Quick DASH values were not available for the initial visit.

#### Sensory symptoms

Current and mean pain intensity during the week prior to examination was measured with a NRS (0 = no pain, 10 = maximum pain imaginable). The character of pain as well as other sensory symptoms were assessed using a multiple-choice list of descriptions including choices for continuous, intermittent and/or orthostatic pain, lancinating pain, pain during movements and no pain (spontaneous pain) as well as presence of pain upon cold and/or warm exposure, touching the skin or slight pressure on the finger joints, presence of pricking or tingling, numbness and/or increased pain perception upon slightly painful stimuli.

#### Motor symptoms

Subjective impairment of motor function was assessed using a multiple-choice list of descriptions including choices for presence of reduced muscle force, joint stiffness, impaired use of function, muscular atrophy, involuntary tremor or malposition (dystonia) of the affected extremity as well as use of extremity only possible under vision. Quantification of function impairment was performed using the task-specific scale [23]: Patients were instructed to choose five activities they had regularly executed prior to the onset of CRPS, but which now were difficult to perform due to the pain. The current capability to perform each activity was then rated on a NRS where 0 = no capability at all and 10 = normal performance of activity. The mean estimation of the five different activities was used to quantify motor impairment.

#### Autonomic symptoms

Patients' perception of the presence of different autonomic symptoms was assessed using a multiple-choice list of descriptions including choices for presence of changes of complexion and/or hair growth, warmer/colder extremity compared to the contralateral extremity, continuous/intermittent edema, increased/decreased sweating and accelerated/decelerated nail growth.

### Investigation of clinical signs

#### Sensory signs

All patients were examined in the most painful area on the affected and corresponding area of the contralateral hand. Quantitative sensory testing (QST) was performed according to the protocol of the German Research Network on Neuropathic Pain which has been described in detail by Rolke et al. [9]. The protocol includes the investigation of mechanical detection (MDT) and vibration detection threshold (VDT) representing the function of large myelinated Aβ-fibers or central pathways, cold detection (CDT), cold pain (CPT), warm detection (WDT) and heat pain threshold (HPT), presence of paradoxical heat sensations (PHS), thermal sensory limen (TSL), mechanical pain threshold (MPT), mechanical pain sensitivity (MPS), wind-up ratio (WUR) and pressure pain threshold (PPT) representing small fiber function (Aδ- or C-fibers) or central pathways as well as presence of dynamic mechanical allodynia (DMA).

In short, thermal detection and thermal pain thresholds were analyzed using a thermode (TSA 2001-II; Medoc, Israel; contact area 7.84cm2) with a baseline temperature of 32°C. To obtain thresholds, the temperature of the thermode was set to increase or decrease at 1°C/s and was terminated when the patient pressed a button. To examine PPT, a spring-loaded pressure threshold (FDN200, Wagner Instruments, USA) was applied to the thenar with a slowly increasing stimulus ramp (50 kPa/s). For assessment of MPT and MPS, pinprick stimuli with fixed stimulus intensities (8, 16, 32, 64, 128, 256, 512 mN; The PinPrick; MRC Systems GmbH, Germany) were used. MPT describes the threshold for pinprick pain, MPS indicates whether hyper- or hypoalgesia exists in the suprathreshold range. The perceived magnitude of pain to a series of pinprick stimuli (pinprick force: 256 mN, repeated 10 times at a 1/s rate on separate spots within a small area of about 1 cm^2^) was compared to a single pinprick stimulus of the same force and defined WUR. WUR was not calculated if the first (single) stimulus was rated NRS 0/100 in more than three assessments, and in this case was handled as missing data. MDT was tested with standardized von-Frey-hairs (Optihair2-Set, Marstock Nervtest, Germany) exerting a force between 0.25 and 512 mN. VDT was measured using a Rydell-Seiffert tuning fork (64Hz, 8/8 scale). DMA was assessed using three different stimuli: a cotton wisp, a cotton wool tip fixed to an elastic strip and a soft brush and the subjects were asked to rate the pain on a 0/100 NRS.

#### Motor signs

Measurement of an individual's ability to tap fingers is an important method of assessing neuromuscular integrity [24]. Finger tapping is widely applied in clinical settings for Parkinson's disease as the rhythm of the dominant hand finger movements acts as an efficient index for evaluation of brain motor function [25]. The patients tapped alternatingly on two buttons (distance 20 cm) with the index finger by using the whole upper extremity [26]. The subjects were instructed to perform as many taps as possible within 10s. Each task was repeated two times for each hand and mean of the number of taps on one extremity defined as tapping score. The percentage deviation between affected and contralateral extremity is used for evaluation of bradykinesia. As the investigation of finger tapping was not part of the protocol of the German Research Network on Neuropathic Pain, values were not available for the initial visit.

For measurement of range of motion the distance of fingertips of dig 2-3-4-5 to the palmar side of the hand were measured in centimeters according to Geertzen et al [27]. The finger-palmar distance was then defined as the mean of these measurements.

#### Autonomic signs

Autonomic signs (hyperhidrosis, hipohydrosis, edema, trophic changes of skin, hypertrichosis, accelerated/ decelerated nail growth) present on physical examination were recorded.

Skin temperature was assessed manually on the testing area as well as on the finger pulps in digits one through five with an infrared thermometer (IR Thermometer, IR-1000 L, Voltcraft, Hirschau, Germany). For skin temperature of the finger tips the mean of the five measurements of each hand was taken.

Upon follow-up, long-term skin temperature was assessed for at least 8h and during the night with small loggers affixed to the small fingers of both hands. Loggers measured temperature every min (Kooltrak, Geisenheim, Germany). The small finger was chosen in order to reduce influences of movement or activities of fingers on skin temperature. Analysis was made according to Krumova et al. [28]. Mean and absolute side differences in skin temperature and the percentage of assessed time when the test side was warmer or colder than 2°C was calculated. The oscillation number of more than 2°C was determined separately for each hand and a ratio between the frequency of oscillations that occurred on the test and control side was analyzed as well as a coefficient of determination of the individual regression equation, a parameter used to describe a-synchronicity between both sides [28].

### Estimation of CRPS severity

In order to quantify severity of CRPS, the CRPS severity score was used. This score is a measure to summarize the different clinical symptoms that characterize CRPS (sensory, vasomotor, sudomotor, and motor/trophic disturbances) into a clinically feasible severity score [29]. Higher scores have been demonstrated to be associated with higher clinical pain intensity, distress and functional impairments as well as greater bilateral temperature asymmetry and thermal perception abnormalities [29].

### Data evaluation

Statistical comparison of QST data was made to a reference data base of healthy controls [9]. All patient data were normalized to the respective gender and age group of the healthy controls and z-values calculated (z = (individual value–mean_data base_) / SD_data base_). Z-scores above “0” indicate hyperfunction, i.e. patients are more sensitive to the tested parameter compared to controls (lower thresholds), whereas Z-scores below “0” indicate hypofunction and therefore a loss of or lower sensitivity of the patient compared to controls (higher thresholds). Both, z-values out of the 95% confidence interval (absolute abnormal value) and a difference of more than two standard deviations in the z-scores between affected and contralateral extremity (abnormal side-to-side difference) were considered as abnormal. Wilcoxon test was used for calculation of intragroup differences between test and contralateral side as well as between first and second measurement. Linear relationships were assessed with Spearman’s rank test. Single tailed Chi^2^-test was used to test whether abnormal values were more frequent in patients than in healthy controls. P < 0.05 was considered statistically significant.

## Results

### Characteristics of patients

No patient had abnormalities besides residues of CRPS upon neurological examination. Characteristics of patients are shown in **Table 2.** 7 (36.8%) patients had a left-sided, 12 (63.2%) a right-sided CRPS. In 8 (42.1%) patients, 3-phase-bone-scintigraphy was performed with a characteristic result for CRPS [30]. Most common comorbidities were arterial hypertension (n = 7, 36.8%), hypothyroidism (n = 2, 10.5%) and hyperthyroidism (n = 2, 10.5%). 11 (57.9%) patients were smokers. Sleep disturbances were described by 6 patients (31.6%).

**Table 2:** Characteristics of patients (n = 19).

	Data ± SD
**Mean age (range) [years]**	58.1 ± 13.8 (19–81)
**Females**	15 (79%)
**Time between iniciating event and complex regional pain syndrome**	
< 1 week	10 (52.6%)
< 1 month	1 (5.3%)
< 2 months	6 (31.6%)
> 2 months	2 (10.5%)
**Iniciating event**	
***with surgery***	13 (68.4%)
Fracture	8 (42.1%)
Soft tissue injury	3 (15.8%)
Joint injury	2 (10.5%)
***without surgery***	6 (31.6%)
Fracture	3 (15.8%)
Soft tissue injury	3 (15.8%)
Joint injury	0
**Time between onset of CRPS and examination at visit 1 (range) [months]**	5.7 ± 8.3 (1–33)
Acute CRPS (< 6 months)	17
Chronic CRPS (≥ 2 years)	2
**Time between visit 1 and follow-up (range) [months]**	36 ± 10.6 (16–53)
**Ongoing pain medication at follow-up**	4 (21.1%)
Non-steroidal anti-inflammatory drugs	2 (10.5%)
Antidepressants	1 (5.3%)
Anticonvulsants	1 (5.3%)
Low potent opioids	2 (10.5%)
High potent opioids	1 (5.3%)
**Concomitant treatment at follow-up**	2 (10.5%)
Physiotherapy	2 (10.5%)
Occupational therapy	0
Psychotherapy	0
Transcutaneous electrical nerve stimulation	0
Interventional treatment (sympathetic blocks, ganglionic local opioid analgesia)	0
Invasive treatment (spinal cord stimulation, deep brain stimulation, neurodestructive procedures)	0

### Clinical presentation of CRPS upon follow-up

Mean time between visit 1 and the follow-up examination was 36 ± 10.6 months (range 16–53 months). In contrast to first assessment, Budapest criteria were only fulfilled by 13 (68.4%) of patients on follow-up examination (p < 0.05, **Table 3**). Two patients (10%) reported worsening, one (5%) no change, but 16 (84%) subjective improvement of symptoms since first assessment (mean improvement 5.4 ± 5.5 NRS). Although CRPS severity score improved on follow-up examination compared to first assessment (8.2 ± 2.6 vs 9.8 ± 1.9, p < 0.05), only two patients (10%) reported no impairment of daily life, whereas all other patients still suffered from mild (n = 5; 26.3%), moderate (n = 7; 36.8%) or even severe (n = 5; 26.3%) impairment of daily life. Approximately one fourth (26.3%) of patients was still unable to work.

**Table 3:** Signs and symptoms including Budapest criteria at visit 1 and follow-up examination.

	Symptoms		Signs					
Category	Visit 1	Follow-up	Visit 1	Follow-up	p symptoms upon 1./follow-up visit	p signs upon 1./follow-up visit	p signs vs symptoms upon 1. visit	p signs vs symptoms upon follow-up
**Pain**	**19 (100%)**	**16 (84.2%)**			**n.s.**			
Continuous pain	16 (84.2%)	7 (36.8%)			**<0.01**			
Intermittend pain	0	7 (36.8%)			**<0.05**			
Pain at orthostatic conditions	15 (79.0%)	4 (21.1%)			**<0.01**			
Pain upon movement	19 (100%)	1 (5.3%)			**<0.01**			
Pain attacks	5 (26.3%)	8 (42.1%)			n.s.			
**Sensory abnormalities**	**17 (89.5%)**	**17 (89.5%)**	**15 (79%)**	**15 (79%)**	n.s.	n.s.	n.s.	n.s.
Tingling paraesthesia^a^	3 (15.8%)	11 (57.9%)			**<0.01**			
Numbness^a^	10 (52.6%)	10(52.6%)	6 (31.6%)	6 (31.6%)	n.s.	n.s.	n.s.	n.s.
***Mechanical Allodynia***	12 (63.2%)	10 (52.6%)	5 (26.3%)	2 (10.5%)	n.s.	n.s.	**<0.05**	**<0.01**
***Hyperalgesia (all)***	9 (47.4%)	14 (73.7%)	11 (57.9%)	14 (73.7%)	n.s.	n.s.	n.s	n.s.
Cold Hyperalgesia	1 (5.3%)	9 (47.4%)	7(36.8%)	10 (52.6%)	**<0.01**	n.s.	**<0.05**	n.s.
Heat Hyperalgesia	2 (10.5%)	5 (26.3%)	7 (36.8%)	10 (52.6%)	n.s.	n.s.	n.s.	n.s.
Mechanical hyperalgesia	8 (42.1%)	10 (52.6%)	19 (100%)	12 (63.2%)	n.s.	**<0.01**	**<0.01**	n.s.
**Autonomic abnormalities**								
***Vascular disorders***	**14 (73.7%)**	**14 (73.7%)**	**14 (73.7%)**	**14 (73.7%)**	n.s.	n.s.	n.s.	n.s.
Skin temperature differences	9 (47.4%)	10 (52.6%)	8 (57.1%)*	8 (42.1%)	n.s.	n.s.	n.s.	n.s.
Changes of skin color	12 (63.2%)	12 (63.2%)	11 (57.9%)	10 (52.6%)	n.s.	n.s.	n.s.	n.s.
***Sudomotor/ Edema***	**19 (100%)**	**13 (68.4%)**	**18 (94.7%)**	**4 (21.1%)**	**<0.05**	**<0.01**	n.s.	**<0.01**
***Edema (all)***	18 (94.7%)	11 (57.9%)	17 (89.5%)	2 (10.5%)	**<0.01**	**<0.01**	n.s.	**<0.01**
Intermittend Edema	3 (15.8%)	9 (47.4%)	0	0	**<0.05**	n.s.	n.s.	**<0.01**
Edema at rest	15 (79%)	2 (10.5%)	17 (89.5%)	2 (10.5%)	**<0.01**	**<0.01**	n.s.	n.s.
***Sudomotor abnormalities (all)***	11 (57.9%)	7 (36.8%)	6 (42.1%)	1 (5.3%)	n.s.	**<0.05**	n.s.	**<0.05**
Hyperhidrosis	9 (42.1%)	5 (26.3%)	5 (26.3%)	0	n.s.	**<0.05**	n.s.	n.s.
Hypohidrosis	3 (15.8%)	2 (10.5%)	2 (10.5%)	1 (5.3%)	n.s.	n.s.	n.s.	n.s.
**Motor/trophic**								
***Trophic symptoms/signs (all)***	11 (57.9%)	6 (31.6%)	5 (26.3%)	1 (5.3%)	n.s.	n.s.	**<0.05**	**<0.05**
Trophical changes of skin	0	0	0	0	n.s.	n.s.	n.s.	n.s.
Hypertrichiosis	4 (21.1%)	3 (15.8%)	4 (21.1%)	1 (5.3%)	n.s.	n.s.	n.s.	n.s.
Increased/ decreased growth of finger-nails	11 (57.9%)	5 (26.3%)	2 (10.5%)	0	**<0.05**	n.s.	**<0.01**	n.s.
***Motor symptoms/signs (all)***	**14 (73.7%)**	**17 (89.5%)**	**17 (89.5%)**	**13 (68.4%)**	n.s.	n.s.	n.s.	n.s.
Reduced strength	10 (52.6%)	16 (84.2%)	10 (52.6%)	6 (31.6%)	**<0.05**	n.s.	n.s.	**<0.01**
Tremor	1 (5.3%)	5 (26.3%)	2 (10.5%)	2 (10.5%)	n.s.	n.s.	n.s.	n.s.
Dystonia	3 (15.8%)	1 (5.3%)	3 (15.8%)	1 (5.3%)	n.s.	n.s.	n.s.	n.s.
Range of motion	6 (31.6%)	15 (79%)	12 (63.2%)	10 (52.6%)	**<0.01**	n.s.	n.s.	n.s.
Muscle atrophie^a^	0	6 (31.6%)	0	5 (26.3%)	n.s.	n.s.	**<0.05**	**<0.05**
Stiffness of joints^a^	6 (31.6%)	15 (79%)	7 (36.8%)	5 (26.3%)	**<0.01**	n.s.	n.s.	**<0.01**
Decreased serviceability^a^	13 (68.4%)	16 (84.2%)	13 (68.4%)	5 (26.3%)	n.s.	n.s.	n.s.	**<0.01**
**Budapest criteria confirmed**	19 (100%)	13 (68.2%)	**<0.05**					

Symptoms are phenomenons told by the patients (subjective), signs are phenomenons found upon examinations by the investigator. Numbness (sign): Abnormal loss of mechanical detection upon QST. Allodynia (symptoms): Agreed with the answer “pain caused by touch”. Allodynia (signs): Abnormal DMA upon QST. Mechanical hyperalgesia (symptoms): Agreed with the answer „increased pain by mechanical stimulation “of the clinical questionnaire. Mechanical hyperalgesia (signs): Presence of at least one abnormal parameter in PPT, MPS or MPT upon QST. Vascular abnormalities: Change of skin color and/ or skin temperature differences of tested limb and contralateral side. Change of skin color is a livide or red coloration of the skin compared to the contralateral limb. Warm/ less warm limb (signs): skin temperature difference ≥1°C compared to the contralateral limb. Range of motion was found by determining the distance of fingertips to palm and defined as affected at a distance > 0 cm.

a: Symptoms/signs were not included for evaluation of Budapest criteria.

* n was 18 for measurement of skin temperature upon visit 1.

### Questionnaires

#### Leeds Assessment of Neuropathic Symptoms and Signs (LANSS)

Total score upon LANSS was reduced in follow-up compared to first measurement (11.6 ± 7.5, range 8–24 vs 18.4 ± 4.9, range 0–24, p < 0.005). At the first examination 17 patients (89%) and at the follow-up examination 12 patients (63.1%) had a score ≥ 12 suggesting that neuropathic mechanisms are likely to be contributing to the patients’ pain.

#### Neuropathic Pain Scale (NPS)

In one patient, NPS was not available. 12 patients (66.6%) demonstrated a reduced total score in NPS upon follow-up, whereas in five (27.7%) NPS total score increased. Overall, there was a trend towards a reduction of NPS total score upon follow-up examination compared to first visit (33.4 ± 21.8 vs 42.6 ± 18.8, p ns). In particular, a decrease of felt pain intensity for heat (2.7 ± 3.1 vs 5.1 ± 2.9, p < 0.05), unpleasantness (4.6 ± 2.7 vs 6.4 ± 2.8, p < 0.05) and superficial pain (2.9 ± 2.1vs 6.0 ± 2.7, p < 0.05) was described. The other parameters including pain intensity for pricking, dullness, cold, sensitivity, itching, or deepness did not differ between the two measurements.

#### German Multidimensional Pain Inventory (MPI)

Upon MPI less disability (2.3 ± 1.6 vs 4.3 ± 1.1, p < 0.05) as well as an improvement of social activity (2.5 ±1.2 vs 1.7 ± 1.1, p < 0.05) and daily in- (4 ± 1.2 vs 2.3 ± 1.4, p < 0.05) and outdoor (1.4 ± 1.6 vs 0.6 ± 0.9, p < 0.05) activities were reported in follow-up compared to first measurement. Social support (2.6 ± 2.1 vs 5.0 ± 3.3, p < 0.01) as well as punishing response (0.7 ± 1.2 vs 1.4 ± 1.6, p < 0.05) were less on follow-up compared to first visit, whereas solicitous response increased (3.8 ± 1.4 vs 1.9 ±1.3, p < 0.05).

#### Disabilities of the Arm, Shoulder and Hand (Quick-DASH)

Quick-DASH was not available for the first visit. Only one patient showed normal Quick-DASH values upon follow-up (normal values: 4.55 for females (44–54 years); 6.82 for males (55–64 years). Mean Quick-DASH score upon follow-up examination was 41.4 ± 5.2 (range 4.6–75) suggesting considerable impaired function of the affected upper extremity.

### Sensory symptoms

#### Pain

At the follow-up examination, 16 patients (84.2%) still reported presence of pain, although the number of patients with continuous pain was considerably lower compared to the first examination (84.2% vs 36.8%, p < 0.01), whereas the number of patients with intermittent pain was higher (0% vs 36.8%, p < 0.05, **Table 3**). Current and mean pain intensity during the last 7 days were reduced in follow-up compared to first examination (2.5 ± 2.2 vs 5.4 ± 3.2, p < 0.001 and 3.1 ± 2.5 vs 5.6 ± 3.0, p < 0.001).

#### Other sensory symptoms

Frequencies of positive and negative sensory signs and symptoms are shown in **Table 3**. Reports for tingling paraesthesia and for cold hyperalgesia were higher upon follow-up compared to first visit **(p<0.01, Table 3**). Compared to subjective reported symptoms, the frequency of mechanical allodynia upon QST was considerably lower at both examinations whereas frequencies for thermal and mechanical hyperalgesia were higher or showed a trend towards higher values upon QST compared to subjective reported symptoms (**Table 3**).

### Sensory signs upon Quantitative Sensory Testing (QST)

#### Frequencies of abnormal values on the ipsilateral side–gain of function

Increased sensitivity for painful thermal and mechanical stimuli as well as dynamic mechanical allodynia and PHS on the affected side was more frequent in CRPS patients compared to controls during visit 1 and follow-up examination (**Table 4**). Frequency of mechanical pain sensitivity for blunt pressure decreased, but increased for pinprick stimuli in follow-up examination compared to visit 1. 12/19 patients (63%) demonstrated an increase in sensitization for at least one thermal or mechanical pain stimulus (**Table 5**). Mechanical pain sensitivity (MPS) on the affected extremity was higher, the higher mean pain intensity (R = 0.6, p < 0.001) and the higher CRPS severity score (R = 0.64, p < 0.005) at follow-up examination were.

**Table 4:** Frequencies of abnormal pathological values (including abnormal side-to-side differences) in CRPS-I.

	**Healthycontrols**	**Affected extremity**			**Contralateral extremity**				
**Gain**	**[n = 180]**	**visit 1[n = 19][WUR n = 15]**	**Follow-up[n = 19][WUR n = 15]**	**P[visit 1 vs follow-up]**	**visit 1[n = 19][WUR n = 15]**	**Follow-up[n = 19][WUR n = 15]**	**P[visit 1 vs follow-up]**	**P [affected vs contralateral visit 1]**	**P [affected vs contralateral follow-up]**
**CDT**	4 (2.3%)	0 (0.0)	0 (0.0%)	n.s.	1 (5.3%)	0 (0.0%)	n.s.	n.s.	n.s.
**WDT**	11 (6.1%)	0 (0.0%)	3 (15.8%)	n.s.	0 (0.0%)	1 (5.3%)	n.s.	n.s.	n.s.
**TSL**	9 (5%)	1 (5.3%)	1 (5.3%)	n.s.	0 (0.0%)	0 (0.0%)	n.s.	n.s.	n.s.
**CPT**	8 (4.5%)	7 (36.8%)**	10 (52.6%)**	n.s.	2 (10.5%)	8 (42.1%)**	**<0.05**	n.s.	n.s.
**HPT**	7 (3.9%)	7 (36.8%)**	10 (52.6%)**	n.s.	0 (0.0%)	3 (15.8%)*	n.s.	**<0.01**	**<0.05**
**PPT**	10 (5.6%)	19 (100%)**	3 (15.8%)	**<0.01**	3 (15.8%)	0 (0.0%)	n.s.	**<0.01**	n.s.
**MPT**	6 (3.3%)	1 (5.3%)	6 (31.6%)**	**<0.05**	0 (0.0%)	4 (21.1%)**	n.s.	n.s.	n.s.
**MPS**	9 (5%)	8 (42.1%)**	8 (42.1%)**	n.s.	2 (10.5%)	7 (36.8%)**	n.s.	**<0.05**	n.s.
**WUR**	13 (7.2%)	1 (6.7%)	2 (13.3%)	n.s.	0 (0.0%)	0 (0.0%)	n.s.	n.s.	n.s.
**MDT**	11 (6.2%)	1 (5.3%)	1 (5.3%)	n.s.	0 (0.0%)	0 (0.0%)	n.s.	n.s.	n.s.
**VDT**	12 (6.7%)	1 (5.3%)	0 (0.0%)	n.s.	0 (0.0%)	0 (0.0%)	n.s.	n.s.	n.s.
**PHS**	0 (0%)	1 (5.3%)*	5 (26.3%)**	n.s.	0 (0.0%)	4 (21.1%)**	n.s.	n.s.	n.s.
**DMA**	2 (1.1%)	5 (26.3%)**	2 (10.5%)**	n.s.	0 (0.0%)	0 (0.0%)	n.s.	**<0.05**	n.s.
	**Healthy controls**	**Affected extremity**			**Contralateral extremity**				
**Loss**	**[n = 180]**	**visit 1[n = 19][WUR n = 15]**	**Follow-up[n = 19][WUR n = 15]**	**P[visit 1 vs follow-up]**	**visit 1[n = 19][WUR n = 15]**	**Follow-up[n = 19][WUR n = 15]**	**P[visit1 vs follow-up]**	**P [affected vs contralateral visit 1]**	**P [affected vs contralateral follow-up]**
**CDT**	12 (6.7%)	6 (31.6%)**	3 (15.8%)	n.s.	0 (0.0%)	0 (0.0%)	n.s.	**<0.05**	n.s.
**WDT**	13 (7.2%)	5 (26.3%)**	4 (21.1%)*	n.s.	3 (15.8%)	6 (31.6%)**	n.s.	n.s.	n.s.
**TSL**	10 (5.5%)	5 (26.3%)**	3 (15.8%)	n.s.	0 (0.0%)	2 (10.5%)	n.s.	**<0.05**	n.s.
**CPT**	8 (4.4%)	2 (10.5%)	1 (5.3%)	n.s.	0 (0.0%)	0 (0.0%)	n.s.	n.s.	n.s.
**HPT**	5 (2.8%)	1 (5.3%)	1 (5.3%)	n.s.	0 (0.0%)	0 (0.0%)	n.s.	n.s.	n.s.
**PPT**	6 (3.4%)	0	8 (42.1%)**	**<0.01**	4 (21.1%)**	13 (68.4%)**	**<0.01**	n.s.	n.s.
**MPT**	9 (5%)	1 (5.3%)	2 (10.5%)	n.s.	0 (0.0%)	0 (0.0%)	n.s.	n.s.	n.s.
**MPS**	4 (2.2%)	1 (5.3%)	1 (5.3%)	n.s.	3 (15.8%)**	2 (10.5%)*	n.s.	n.s.	n.s.
**WUR**	5 (2.8%)	1 (6.7%)	3 (20%)**	n.s.	1 (6.7%)	1 (6.7%)	n.s.	n.s.	n.s.
**MDT**	11 (6.1%)	6 (31.6%)**	6 (31.6)**	n.s.	1 (5.3%)	0 (0.0%)	n.s.	**<0.05**	**<0.05**
**VDT**	2 (1.1%)	8 (42.1%)**	6 (31.6%)**	n.s.	3 (15.8%)**	2 (10.5%)**	n.s.	n.s.	n.s.

CDT: cold detection threshold, WDT: warm detection threshold, TSL: thermal sensory limen, CPT: cold pain threshold, HPT: heat pain threshold, PPT: Pressure pain threshold, MPT: mechanical pain threshold, MPS: Mechanical pain sensitivity, WUR: wind-up ratio, MDT: Mechanical detection threshold, VDT: vibration detection threshold, DMA: dynamic mechanical allodynia, PHS; paradoxical heat sensations.

* p < 0.05 compared to healthy controls

** p < 0.01 compared to healthy controls

**Table 5:** Absolute abnormal values upon QST at follow-up and their change compared to visit 1.

Patient	QST	SI	Pain	NPS	CRPS severity score	Motor	Autonomic	Result
1	PHS contralateral +, WDT–bil. (↓), VDT–bil. (↓), MPS + bil. (↑), PPT–bil. (↓),	-6	Ongoing, NRS 5 (↑)	39 (↑)	12 (↑)	+	+	Increasing bilateral loss and sensitization (MPS); pain-induced hypoaesthesia?
2	VDT ipsilateral–(↓), CPT + bil. (↑), HPT + bil. (↑), MPS + bil. (↑), PPT–bil- (↓)	6.5	Ongoing, NRS 5.5 (↓)	52 (↑)	9 (↓)	+	+	Increasing bilateral sensitization despite pain reduction and improvement
3	CPT + bil. (↔), HPT bil. + (↑), MPS + bil. (↑)	10	Intermittent, NRS 3.5 (↓)	15 (↓)	12 (↓)	+	+	Increasing bilateral sensitization despite pain reduction and improvement
4	HPT normalized ipsilateral, MPT contralateral + (↑), WUR contralateral + (↑), PPT–bil. (↓)	10	Intermittent, NRS 2.5 (↓)	68 (↑)	8 (↓)	+	+	Increasing contralateral sensitization despite pain reduction
5	CPT ipsilateral + (↔), HPT ipsilateral + (↔), MPT contralateral + (↑), MPS + bil. (↑)	0	Ongoing,NRS 2 (↔)	13 (↓)	6 (↓)	+	+	Increasing bilateral sensitization
6	WDT ipsilateral–(↔), TSL ipsilateral–(↔), MDT ipsilateral–(↔), VDT normalized ipsilateral, CPT ipsilateral + (↑), MPS + bil. (↑)	5	Ongoing,NRS 7.5 (↔)	44 (↓)	9 (↑)	+	+	Continuing ipsilateral loss, increasing bilateral sensitization
7	WDT contralateral–(↔), VDT normalized bil., WUR ipsilateral + (↑), PPT ipsilateral–(↓)	8	Intermittent,NRS 1 (↓)	11 (↓)	3 (↓)	+	+	Ipsilateral hints for increasing central sensitization (WUR)
8	WDT–ipsilateral (↔), CDT/TSL/MDT normalized ipsilateral, DMA + ipsilateral (↔), MPT + bil. (↔),	2	Ongoing,NRS 2 (↓)	56 (↓)	10 (↔)	+	+	Normalization of detection thresholds, continuing central sensitization
9	WDT contralateral–(↓), TSL contralateral–(↓), CPT + ipsilateral (↔), CPT + contralateral (↑), HPT normalized ipsilateral, MPS + bil. (↑)	6.5	Intermittent,NRS 5 (↓)	30 (↓)	12 (↓)	+	+	Increasing thermal loss contralateral, ongoing sensitization ipsilateral, increasing sensitization contralateral despite improvement
10	DMA and PPT normalized ipsilateral	9	No pain	0 (↓)	8 (↔)	+	+	Complete relief of pain, no QST abnormalities
11	WDT ipsilateral–(↓), CPT ipsilateral + (↔), CPT contralateral + (↑), HPT ipsilateral + (↔), MPS + bil. (↔), PPT–bil. (↓)	8	Intermittent,NRS 4 (↓)	20 (↓)	9 (↑)	+	+	Ongoing thermal loss ipsilateral and sensitization bilaterally, increasing contralateral sensitization
12	CDT–ipsilateral (↓), WDT–contralateral (↔), TSL—contralateral (↓), CPT contralateral + (↑), HPT + ipsilateral (↔), MPT + ipsilateral (↑), PPT–bil. (↓)	7	No pain	n.a.	7 (↓)	+	+	Increasing loss and sensitization bilaterally despite complete relief of pain
13	CDT and MDT ipsilateral normalized, WDT–ipsilateral (↔), WDT contralateral (↓), PPT–bil. (↓)	10	No pain	n.a.	2 (↓)	+	+	No sensitization, normalization of cold and detection thresholds with pain relief, but not for warm detection thresholds
14	DMA normalized ipsilateral, MPS + ipsilateral (↔), PPT–bil. (↓)	5	Intermittent,NRS 3 (↓)	39 (↓)	8 (↓)	+	+	Improvement of sensitization with pain reduction
15	VDT–bil. (↔), CPT + ipsilateral (↑), HPT + ipsilateral (↑)	5	Intermittent,NRS 5 (↔)	34 (↑)	9 (↔)	+	+	Ongoing bilateral loss and increasing ipsilateral sensitization with steady pain
16	VDT normalized ipsilateral, CPT + bil. (↑), HPT + ipsilateral (↔), MPT + ipsilateral (↑), PPT–bil. (↓)	10	Intermittent, NRS 1 (↓)	25 (↓)	8 (↑)	+	+	VDT normalized ipsilateral with reduction of pain, but increased bilateral sensitization
17	HPT and DMA normalized ipsilateral, PPT–bil. (↓)	-10	Ongoing, NRS 7 (↓)	79 (↑)	7 (↓)	+	+	No loss, decreasing sensitization
18	MDT normalized ipsilateral, VDT normalized bilateral, CDT–ipsilateral (↔), TSL–ipsilateral (↔), CPT + bil. (↑), HPT + ipsilateral (↑), MPT + bil. (↑), MPS + bil. (↑)	7	Ongoing,NRS 5.5 (↓)	34 (↓)	9 (↓)	+	+	Normalization of mechanical loss bilaterally, but ongoing ipsilateral thermal loss and increasing bilateral sensitization despite pain reduction
19	CPT + bil. (↑), MPT + ipsilateral (↑), PPT–bil. (↓)	9	Intermittent, NRS 1 (↓)	8 (↓)	7 (↔)	+	+	Increasing bilateral sensitization despite pain reduction and improvement

+: gain, -: loss, SI: Subjective Improvement, bil.: bilateral; ↓ loss compared to examination upon visit 1, ↑ gain compared to examination upon visit 1. NRS: Numerical Rating scale (0 = no pain, 10 = maximum imaginable pain), n.a.: not available upon follow-up, CDT: cold detection threshold, WDT: warm detection threshold, TSL: thermal sensory limen, CPT: cold pain threshold, HPT: heat pain threshold, PPT: Pressure pain threshold, MPT: mechanical pain threshold, MPS: Mechanical pain sensitivity, WUR: wind-up ratio, MDT: Mechanical detection threshold, VDT: vibration detection threshold, DMA: dynamic mechanical allodynia, PHS; paradoxical heat sensations.

#### Frequencies of abnormal values on the contralateral side–gain of function

On the contralateral side, a higher frequency for increased sensitivity to painful thermal and mechanical stimuli as well as paradoxical heat sensitivity (PHS) compared to healthy controls was observed in the follow-up examination, but not upon visit 1 (**Table 4**). In contrast to the affected side, cold pain sensitivity of the contralateral side was more frequent in follow-up examination compared to visit 1 (**Table 4**). Furthermore, there was a trend towards increased frequencies for heat and mechanical pain (MPT, MPS) upon follow-up.

#### Frequencies of abnormal values on the ipsilateral compared to the contraleatral side–gain of function

While frequencies for abnormal values upon heat and mechanical pain sensitivity (MPS), hyperalgesia to blunt pressure (PPT) and dynamic mechanical allodynia (DMA) were higher on the affected extremity compared to the contralateral extremity upon visit 1 these differences assimilated upon follow-up. Patients seemed to sensitize in the course of the disease resulting in increased frequencies of abnormal values for thermal and mechanical pain also on the contralateral side (**Table 4**). Besides an increase in ipsilateral sensitization between visit 1 and follow-up, this contralateral increase in sensitization for at least one painful thermal or mechanical stimulus was observed in 12/19 (63%) of patients (**Table 5).** In these, increased sensitivity to cold pain (7/12 patients, 58%) and mechanical pain (MPS: 7/12 patients, 58%; MPT: 3/12 patients, 25%) were the most frequent. Sensitivity for cold pain (R = 0.48, p < 0.05) and mechanical pain (R = 0.48, p < 0.05) on the contralateral extremity were higher, the higher the CRPS severity score at follow-up examination was. Similarly, wind-up ratio on the contralateral extremity (R = 0.5, p < 0.05) was higher, the higher the NPS sum score at follow-up examination was. Thus, overall, increase in sensitization of both, affected and contralateral extremity was associated with a stronger impairment due to CRPS. Interestingly, it was independent from subjective improvement (**Table 5**).

#### Frequencies of abnormal values on the ipsilateral side–loss of function

As already observed by Gierthmühlen et al. (18), loss of detection for thermal and mechanical stimuli was more frequent on the affected extremity in CRPS compared to controls upon visit 1 and follow-up (**Table 4**). On follow-up, however, abnormalities for thermal and mechanical detection were less frequent, but still more common in CRPS than in healthy controls (**Table 4**).

With the exception of loss of sensitivity to blunt pressure stimuli, hypoalgesia to thermal or mechanical stimuli was not very frequent in both visit 1 and follow-up **(Table 4**) and frequencies for painful and non-painful thermal and mechanical loss did not differ between visit 1 and follow-up on the affected extremity.

#### Frequencies of abnormal values on the contralateral side–loss of function

Loss for thermal and mechanical detection on the contralateral extremity upon visit 1 was not as frequent as on the affected extremity, but loss for vibration detection on both visits as well as for warm detection upon follow-up were more frequent compared to controls (**Table 4**). Furthermore, mechanical hypoalgesia to blunt pressure (PPT) and pinprick (MPS) was more frequent on the contralateral extremity compared to healthy controls (**Table 4**).

#### Frequencies of abnormal values on the ipsilateral compared to the contraleatral side–loss of function

Loss for mechanical, but not vibration detection was more frequent on the affected compared to the contralateral extremity upon both visits. Similarly, loss for cold detection and thermal sensory limen was more frequent on the affected extremity upon visit 1. Although not as obvious as for gain, a bilateral loss of detection was observed in 7 (36.8%) patients. 5 (26.3%) demonstrated an increasing loss of detection in follow-up compared to visit 1 with thermal detection thresholds being more affected than mechanical detection thresholds (**Table 5**). In 3 of these patients, the contralateral side was affected. 6 (31.5%) patients showed normalization of mechanical and/ or vibration detection thresholds. Despite this improvement, 5 of them had a stable or only minimally improved loss of thermal detection. Loss of detection was independent from subjective improvement or CRPS severity score.

#### Influence of pain on gain and loss

**Fig 1:**
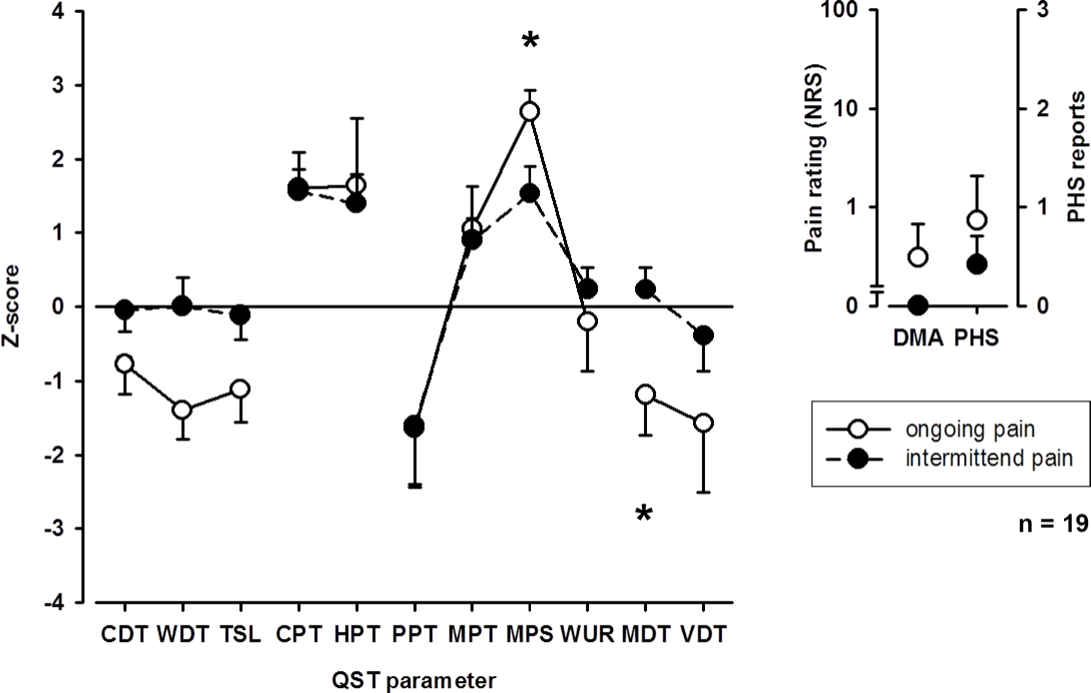
QST Profiles patients with ongoing and intermittent pain. When patients were grouped into those with ongoing or intermittent pain at follow-up examination, patients with ongoing pain showed higher mechanical pain sensitivity as well as a stronger loss for mechanical detection on the affected extremity. CDT: cold detection threshold; WDT: warm detection threshold; TSL: thermal sensory limen; CPT: cold pain threshold; HPT: heat pain threshold; PPT: pressure pain threshold; MPT: mechanical pain threshold, MPS: mechanical pain sensitivity; WUR: wind-up ratio; MDT: mechanical detection threshold; VDT: vibration detection threshold; PHS: paradoxical heat sensitivity; DMA: dynamic mechanical allodynia. * p < 0.05.

When patients were grouped into those with ongoing or intermittent pain at follow-up examination, patients with ongoing pain showed higher mean pain intensity (4.9 ± 2.2 vs 2.1 ± 2.0, p < 0.05) and mechanical pain sensitivity as well as a stronger loss for mechanical detection on the affected extremity. Somatosensory profiles of patients with intermittent and ongoing pain are shown in **Fig 1**. Although patients with a pain reduction between visit 1 and follow-up did not differ in mean pain intensity from those without a pain reduction during both visits (3.0 ± 2.4 vs 3.5 ± 3.1), patients without a pain reduction demonstrated more severe loss of warm detection (-2.2 ± 0.4 vs -0.1 ± 1.2, p < 0.05), but less sensitivity to heat pain (-0.1 ± 1.2 vs 2.0 ± 1.6, p < 0.05).

#### Somatosensory profile upon visit 1

**Fig 2:**
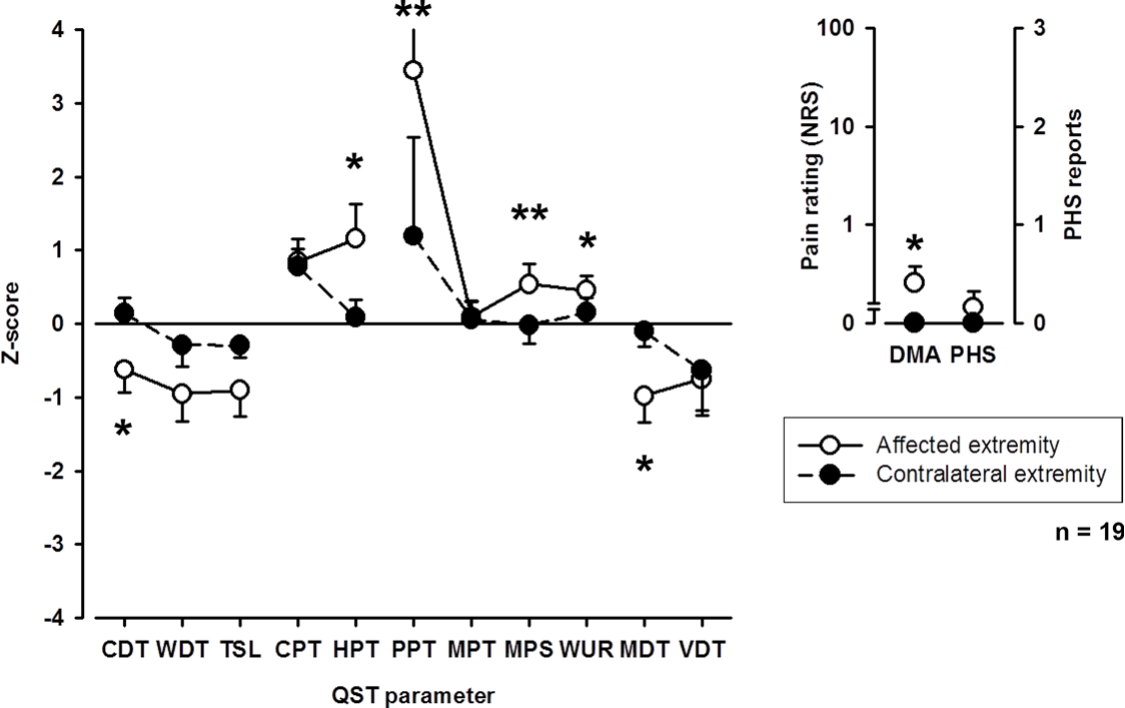
QST profiles of affected and unaffected extremity upon first visit. CDT: cold detection threshold; WDT: warm detection threshold; TSL: thermal sensory limen; CPT: cold pain threshold; HPT: heat pain threshold; PPT: pressure pain threshold; MPT: mechanical pain threshold, MPS: mechanical pain sensitivity; WUR: wind-up ratio; MDT: mechanical detection threshold; VDT: vibration detection threshold; PHS: paradoxical heat sensitivity; DMA: dynamic mechanical allodynia. * p < 0.05, ** p < 0.01.

The somatosensory profile of the affected and corresponding contralateral extremity is shown in **Fig 2**. Compared to healthy controls, CRPS patients were characterized by an increased sensitivity for heat (p < 0.05) and pressure pain (p < 0.01) as well as increased warm and mechanical detection thresholds (p < 0.05) on the affected extremity supporting findings by Gierthmühlen et al. (18). No differences compared to healthy controls were observed on the unaffected contralateral extremity.

Cold and mechanical detection thresholds (MDT) as well as sensitivity for heat and mechanical pain to blunt pressure, pinprick (MPS) and dynamic mechanical allodynia were increased on the affected compared to the corresponding contralateral extremity (**Fig 2**).

#### Somatosensory profile upon follow-up

**Fig 3:**
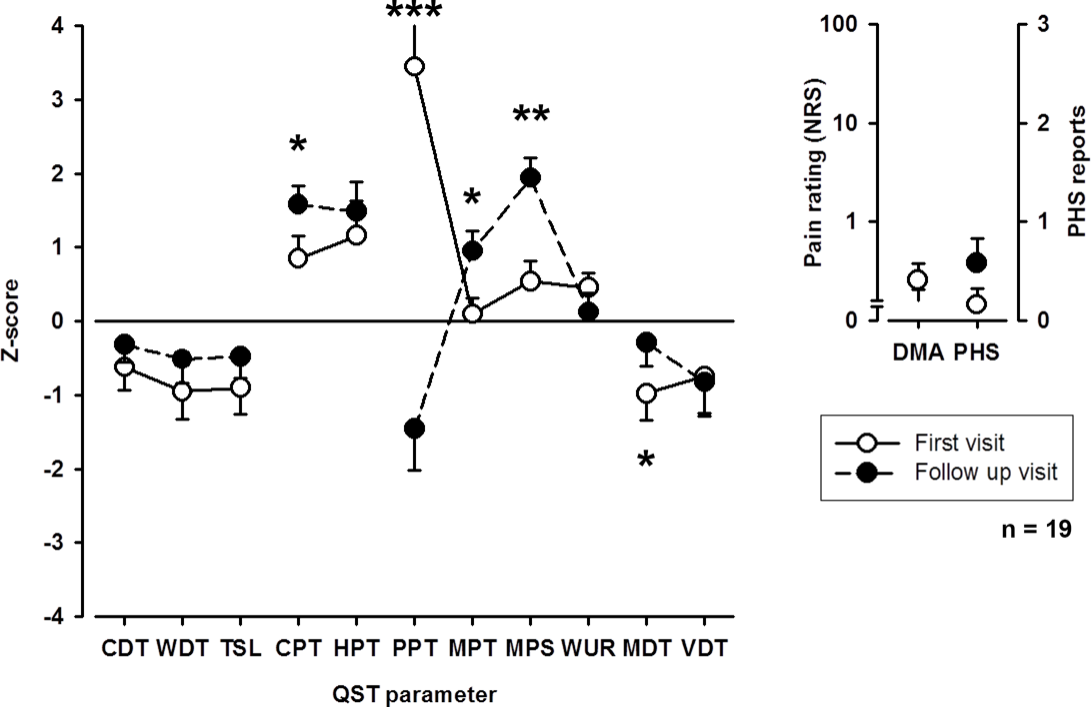
QST profiles of the affected extremity upon first visit and follow-up examination. CDT: cold detection threshold; WDT: warm detection threshold; TSL: thermal sensory limen; CPT: cold pain threshold; HPT: heat pain threshold; PPT: pressure pain threshold; MPT: mechanical pain threshold, MPS: mechanical pain sensitivity; WUR: wind-up ratio; MDT: mechanical detection threshold; VDT: vibration detection threshold; PHS: paradoxical heat sensitivity; DMA: dynamic mechanical allodynia. * p < 0.05, ** p < 0.01, *** p < 0.001.

**Fig 4:**
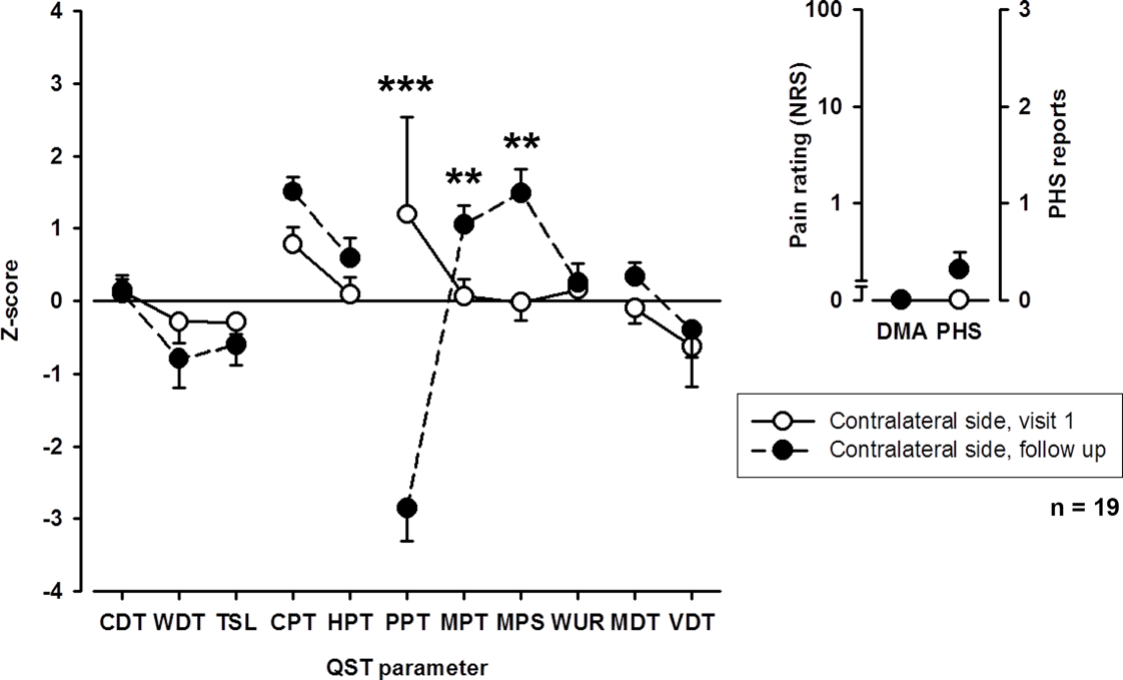
QST profiles of the unaffected (contralateral) extremity upon first visit and follow-up examination. CDT: cold detection threshold; WDT: warm detection threshold; TSL: thermal sensory limen; CPT: cold pain threshold; HPT: heat pain threshold; PPT: pressure pain threshold; MPT: mechanical pain threshold, MPS: mechanical pain sensitivity; WUR: wind-up ratio; MDT: mechanical detection threshold; VDT: vibration detection threshold; PHS: paradoxical heat sensitivity; DMA: dynamic mechanical allodynia. ** p < 0.01, *** p < 0.001.

Upon follow-up, loss of mechanical detection (MDT) on the affected extremity improved (**Fig 3**). Also, thermal detection thresholds showed a trend towards an improvement. Interestingly, however, patients showed an increase in pain sensitivity to cold and pinprick (MPT, MPS) stimuli, whereas sensitivity to blunt pressure (PPT) decreased. This increase in pain sensitivity for thermal and mechanical stimuli, but not for blunt pressure was also observed on the contralateral extremity (**Fig 4**).

Overall, small and large nerve fiber function tended to improve whereas signs for central sensitization increased.

### Motor signs and symptoms

For the presence of motor symptoms subjective description of patients was compared to the objective inspection of the examiner. Most frequent reported motor symptoms upon first visit were reduced strength in 10 (52.6%), decreased serviceability in 13 (68.4%), followed by stiffness of joints and decreased range of motion in 6 (31.6%) patients. Consistent with the abnormal Quick-DASH results functional impairment was rated 4.3 ± 2.4 (range 1–10) on the NRS upon follow-up. Overall, central motor symptoms such as dystonia and tremor were rare symptoms upon both visits. Muscle atrophy was a motor symptom that was only observed upon follow-up (**Table 3**). Whereas patients described a worsening of motor symptoms such as reduced strength, decreased range of motion and stiffness of joints upon follow, the particular motor signs seen by the examiner did not worsen, but even tended to improve (**Table 3**). However, the mean number of finger taps was 32.6 ± 14.6 on the affected extremity (76.5% of unaffected side) and 42.6 ± 15 on the unaffected extremity upon follow-up suggesting a persisting impairment of motor function.

### Autonomic signs and symptoms

#### Skin temperature

Skin temperature did not differ between affected and unaffected extremity upon visit 1 (32.9 ± 2°C on the affected vs 32.7 ± 1.3°C on the unaffected extremity, p n.s.) or follow-up (30.3 ± 2.4°C on the affected vs 29.9 ± 2.6°C on the unaffected extremity, p n.s.) examination.

However, long term skin temperature measurement upon follow-up revealed a pathological increase of cold phases in three (21.4%) patients (23.5%, 28.8% and 18.3% of cold phases, respectively; normal values: < 14.6%) and warm phases in one (7.1%) patient (16% warm phases; normal value: <11.8%) leading to a significantly colder / warmer affected extremity, respectively. Intra-individual correlations and temperature oscillations between affected and contralateral extremity were decreased in 9 (64.3%) and 4 (28.6%) patients, respectively.

#### Other autonomic signs and symptoms

Other autonomic signs and symptoms are summarized in **Table 3**. The most frequent symptom and sign on first visit was an edema (**Table 3**). While edema at rest was the most frequent sign and symptom upon visit 1, an intermittent edema was the most frequent form of edema upon follow- up.

The frequency of sudomotor abnormalities decreased in the course of the disease (**Table 3**). Hyperhidrosis was more frequent than hypohidrosis upon first visit and follow-up. Increased or decreased growth of finger nails was described more frequent upon first visit (57.9%) than upon follow-up (26.3%, **Table 3**). Hypertrichiosis was a rare sign and symptom on both examinations.

### Treatment of CRPS

Approximately half (47.4%) of the patients received a multimodal treatment with a combination of physiotherapy, transcutaneous electrical nerve stimulation, psychotherapy, interventional or pharmacological treatment, the other half was treated with oral pharmacotherapy only (47.4%) and one patient (5.3%) received a single interventional therapy (**Table 6**). The most frequent analgesics used during the entire treatment of CRPS were NSAID in 11 (57.9%), antidepressants (tricyclics) in 10 (52.6%) and anticonvulsants (pregabalin, gabapentin) in 10 (52.6%) patients. Interventional treatment was used in 8 (42.1%) patients. Patients who received only one kind of treatment did not differ from patients with different kinds of treatment with regard to pain, presence of hyperalgesia upon QST or subjective improvement (**Table 6**).

**Table 6:** Treatment of CRPS patients.

**Treatment in patients (No)**	**1**	**2**	**3**	**4**	**5**	**6**	**7**	**8**	**9**	**10**	**11**	**12**	**13**	**14**	**15**	**16**	**17**	**18**	**19**
**Pharmacological Treatment**																			
Antidepressants	1	1	1	0	1	0	1	0	1	0	1	0	0	0	1	0	1	1	0
Anticonvulsants	1	1	0	0	1	1	0	0	1	1	1	0	1	1	0	0	1	0	0
Opioids	1	1	0	0	0	1	0	0	1	0	0	0	0	0	0	0	1	1	1
NSAID	0	1	1	0	1	1	0	1	1	0	1	0	0	1	1	0	1	1	0
**All**	**3**	**4**	**2**	**0**	**3**	**3**	**1**	**1**	**4**	**1**	**3**	**0**	**1**	**2**	**2**	**0**	**4**	**3**	**1**
**Other Noninvasive Treatment**																			
Physiotherapy	0	0	0	0	0	0	0	0	0	0	0	0	0	0	0	1	0	1	0
Psychotherapy	0	0	2	0	0	0	0	0	3	0	0	0	0	0	0	3	0	0	0
TENS	0	0	0	1	0	0	0	0	1	0	0	0	0	0	0	1	0	0	0
**All**	**0**	**0**	**2**	**1**	**0**	**0**	**0**	**0**	**4**	**0**	**0**	**0**	**0**	**0**	**0**	**5**	**0**	**1**	**0**
**Invasive Treatment**																			
Interventional Therapy	1	0	0	2	0	1	0	0	1	0	1	1	0	0	1	2	0	0	0
Other Treatment	0	0	0	4	0	0	0	0	2	0	0	0	0	0	0	4	0	0	0
**All**	**1**	**0**	**0**	**6**	**0**	**1**	**0**	**0**	**3**	**0**	**1**	**1**	**0**	**0**	**1**	**6**	**0**	**0**	**0**
**All Therapies [n]**	**4**	**4**	**4**	**7**	**3**	**4**	**1**	**1**	**11**	**1**	**4**	**1**	**1**	**2**	**3**	**11**	**4**	**4**	**1**
Subjective Improvement [NRS]	-6	6.5	10	10	0	5	8	2	6.5	9	8	7	10	5	5	10	10	7	9
QST LoGa Classification	L3G2	L2G3	L0G3	L0G2	L0G3	L3G2	L0G0	L1G2	L0G3	L0G0	L0G3	L1G3	L1G0	L0G0	L2G1	L0G3	L0G0	L1G3	L0G2
Pain [NRS]	5	5.5	3.5	2.5	2	7.5	1	2	5	0	4	0	0	3	5	1	7	5.5	1

The table shows treatment in the individual 19 patients. Subjective improvement: Improvement/worsening at time of follow-up examination compared to first examination. Antidepressants: Tricyclics. Anticonvulsants: Pregabalin, Gabapentin. Psychotherapy: Behavior therapy. Interventional therapy: Sympathetic blocks. Other treatment: Sympathectomy, spinal cord stimulation, neurodestructive procedures. Subjective improvement: Judgments by NRS in which -10 is the worst and +10 best improvement. QST hyperalgesia upon follow-up examination: LoGa Classification (Maier 2010); L0: no loss of detection, L1: only thermal loss, L2: only mechanical loss, L3: mixed loss of detection, G0: no gain (= no hyperalgesia), G1: only thermal hyperalgesia, G2: only mechanical hyperalgesia, G3: thermal and mechanical hyperalgesia. QST: Quantitative Sensory Testing, TENS: Transcutaneous electrical nerve stimulation, VAS: visual analogue scale, NRS: numerical rating scale.

## Discussion

The present study shows that in the course of the disease (A) CRPS-I patients have an improvement of pain, sudomotor and vasomotor functions, but an ongoing impairment of sensory and motor function still leading to an impairment of daily life activity, (B) demonstrates that despite an improvement of pain and function patients with CRPS show a sensitization of the nociceptive system including the contralateral side and (C) suggests that loss of function in CRPS-I is due to central plasticity induced by activation of the nociceptive system i.e. pain-induced hypoesthesia.

### Signs and symptoms in the course of the disease

A systematic review of cross-sectional as well as pro- and retrospective studies has shown that vasomotor and sudomotor symptoms of CRPS tended to be the most common in the early stages of the disease and were the most likely to resolve [8]. The present study mirrors these results. While sudomotor/edema signs and symptoms were initially present in nearly all patients, they had significantly decreased at the follow-up visit. It is thought that vasomotor and sudomotor symptoms of CRPS are caused by acute inflammatory processes leading to an increased plasma extravasation and vasodilatation. However, while these inflammatory factors contribute to CRPS in the acute phase, they are believed to play a lesser role in later phases of the disease [31]. Indeed, our results demonstrate that inflammatory signs are reduced upon follow-up. Long-term skin temperature measurement revealed an abnormal temperature regulation upon follow-up, in line with a dysfunction of the hypothalamic thermoregulation which has been proposed as a pathophysiological mechanism in CRPS [32].

While sudomotor and vasomotor symptoms tended to improve, long-term follow-up studies have found that motor dysfunction, sensory symptoms and mild pain persisted in CRPS-I [8]. The current study confirms this observation. There was no clear improvement of motor symptoms or signs and even an increase of patients reporting a reduction of strength or range of motion as well as joint stiffness. Furthermore, patients still reported a high function impairment of the affected extremity.

### Sensory signs and symptoms

While most patients still experienced pain at follow-up, its frequency was reduced from continuous to intermittent pain and it was less likely to be triggered by movement or orthostatic conditions with only four patients still needing ongoing pain medication. This reproduces the findings of two previous prospective studies that have measured fairly low pain intensity in CRPS upon follow-up examinations [33, 34]. Despite a reduction in pain intensity, mechanical and thermal pain sensitivity increased upon follow-up on affected and contralateral extremity suggesting a generalized sensitization of the nociceptive system in the course of the disease. Similar contralateral sensory changes in CRPS have been observed in a cross-sectional study [35]. Central sensitization including wind-up mechanisms in the spinal cord, maladaptive neuroplasticity with changes in endogenous pain modulation and reorganization of the somatosensory cortex have been proposed pathophysiological mechanisms in CRPS [2, 31]. The provoking factor for such a generalized sensitization of the nociceptive system could be the continuous nociceptive input in earlier phases of the disease leading to central sensitization and modulatory processes in supraspinal nociceptive centers [36] that might result in a generalized nociceptive facilitation, i.e. a pronociceptive pain modulation profile [37]. This hypothesis is supported by the current study, as the mechanical pain sensitivity on the affected extremity correlated with the mean pain intensity and was further associated with the occurrence of ongoing pain. However, beyond an increased sensitivity to nociceptive stimuli, nociceptive facilitation can also be caused by a reduced endogenous pain inhibition.

Conditioned pain modulation is one well-known endogenous nociceptive modulatory mechanism. It is mediated by the subnucleus reticularis dorsalis in the caudal brainstem and leads to a generalized inhibition of nociceptive spinal cord neurons as a reaction to a painful stimulus [38, 39]. Accordingly, less efficient inhibitory pain modulation has been reported for several pain syndromes [37] and a decreased activity in subnucleus reticularis dorsalis has recently been shown during experimentally induced central sensitization [40]. Furthermore, a downregulation of periaqueductal gray–mediated descending nociceptive inhibition could add to the generalized facilitation of nociceptive stimuli [32]. Supportingly, a recent fMRI study demonstrated that CRPS patients show less activation in the periequaductal gray compared to healthy controls during subsequent painful stimulation of the symptomatic as well as the contralateral asymptomatic extremity [41].

In contrast to thermal and other kinds of mechanical pain sensitivity, pain sensitivity to blunt pressure decreased upon follow-up. It has been proposed that an increased sensitivity to blunt pressure might mirror pathomechanisms of deep-somatic tissue [6]. Supporting this idea, a decreased sensitivity to blunt pressure in CRPS correlates with an increase of periarticular bone turnover in 3-phase-bone-scintigraphy [42]. Usually, there is a normalization of characteristic results in 3-phase-bone-scintigraphy during the course of the disease [30, 43]. Results therefore support the idea, that the increased sensitivity to blunt pressure in CRPS is not a marker for sensitization but rather a hint for an involvement of deep somatic tissue, i.e. an increased bone metabolism, which is thought to correspond to inflammatory processes at the beginning of the disease [44].

We observed a very strong decrease of pain sensitivity to blunt pressure on the affected and contralateral extremity upon follow-up compared to first visit (from hyperalgesic to hypoalgesic values). Although the clear trend of a reduction of pain sensitivity to blunt pressure is obvious, the extent of the reduction on both extremities might indicate a measurement error due to a too fast increase of pressure application upon follow-up. Nevertheless, this does not affect the main results of this study.

### Questionnaires

LANSS, NPS and MPI showed an improvement of sensory symptoms and functionality in the course of the disease. Interestingly, the majority of patients had a LANSS score ≥ 12 at both visits suggesting that neuropathic mechanisms are likely to contribute to the patients’ pain. Moreover, patients showed mean NPS scores of 42.6 and 33.4 at the initial and follow-up visit, respectively, and therefore described distinct pain qualities associated with neuropathic pain [16]. Furthermore, many patients showed somatosensory abnormalities (gain and loss) compatible with neuropathic pain. Nevertheless, the issue as to whether CRPS-I is a neuropathic pain syndrome needs further investigation.

### Loss in CRPS-I–minimal nerve lesion or central plasticity?

It is possible that despite the absence of a major nerve lesion, the patients’ initiating event lead to a minimal nerve injury, which could not be detected in electrophysiology [45], but induced similar mechanisms of gain and loss upon QST as the major nerve lesion in CRPS-II [6]. Indeed, pathological alterations of cutaneous innervation and vasculature have been described in CRPS [45, 46]. Seen in context with the LANSS and NPS scores, this loss could likely be a correlate for a minimal nerve lesion. Moreover, there was an improvement in detection of mechanical and thermal non-painful stimuli on the affected extremity at the follow-up visit, in line with a restoration of nerve function [47]. However, there are several factors that argue against a regenerating minimal nerve lesion: (A) Nerve injuries usually recover in a certain order with complete recovery of mechanical detection only after restoration of pain sensitivity [48] which is in contrast to the findings of QST upon follow-up. Since the mean time between first visit and follow-up was 36 month, it can be assumed that there was sufficient observation time for a possible detection of nerve regeneration. (B) Nerve regeneration cannot explain the observed contralateral changes. (C) The important clinical observation of a characteristic distal spread of signs and symptoms into the affected limb in a glove-like or stocking-like manner in CRPS [49–51] beyond the innervation territories of certain nerves cannot be explained by a minimal nerve lesion.

Rather, the observed association between reduced pain intensity and concomitant improvement in thermal and mechanical detection suggests that loss of function is due to central plasticity induced by activation of the nociceptive system as it has been shown in clinical and experimental pain, that is, pain-induced hypoesthesia [52–55].

### Limitations

When interpreting the results it has to be kept in mind that signs and symptoms of CRPS are often dynamic, i.e. change due to external or internal factors such as pain, environmental temperature, posture, movement etc. This can be a possible explanation for the observed difference between signs and symptoms and influences frequencies of abnormalities. Furthermore, it is probable that patients who completely recovered from CRPS type I will be less likely to take part in the experiment than those who still have symptoms and would like to help researchers to improve knowledge and treatment of CRPS. Therefore, we believe that a selection bias could be an important limitation for the presented results. This study aimed to investigate residues of CRPS in a follow-up after a long time of disease duration. To investigate the fast changes that occur in the course of the disease, further examinations are necessary that repeat investigations in shorter intervals.

### Summary

In conclusion, results suggest central plasticity i.e. pain induced hypoesthesia as underlying mechanism for loss of function in CRPS-I. CRPS-I patients show an improvement of pain and autonomic function in the course of the disease but still a great impairment of sensory and motor function. Furthermore, CRPS-I patients show a sensitization of the nociceptive system including the contralateral side even after successful pain treatment. This sensitization was associated with higher mean pain intensity and the presence of ongoing pain, again indicating central plasticity as one underlying mechanism in CRPS-I.

## Supporting Information

S1 Protocol Study protocol for the follow-up visit (german version). (PDF)

S2 Protocol Study protocol for the follow-up visit (english version). (PDF)
