# Supplementary material for: Sensitization of the Nociceptive System in Complex Regional Pain Syndrome
Source: PLoS One. 2016 May 5;11(5):e0154553. doi: 10.1371/journal.pone.0154553 (PMC4858201; doi:10.1371/journal.pone.0154553)
Supplement: S1 Protocol — (PDF) [file pone.0154553.s001.pdf]

Sensitization of the nociceptive system in complex regional pain syndrome - study protocol (German)

Alle Fragen sollen sich nur auf das CRPS bzw. die erkrankte Extremität beziehen, wenn nicht anders beschrieben!

Alter (Jahre):

Geschlecht

m w

Lokalisation des CRPS:

Left: Right:

Dauer des CRPS (Monate):

Zeit zwischen auslösendem Ereignis/Beginn der Schmerzen und Therapiebeginn

__________________ Monate

< 1 Woche < 1 Monat < 2 Monate > 2 Monate

Dauer zwischen 1. QST-Messung und Telefonkontakt/2. Messung (Monate):

Auslösendes Trauma

Mit oder ohne vorangegangene Operation mit ohne

Engpaßsyndrom	
Fraktur	
Gelenkverletzung	
Gelenk-/Knochenaffektion	
Weichteilerkrankung	
Weichteilverletzung (Distorsion etc.)	
spontan	

Positive Szintigraphie (ggf. vor der 1. QST-Messung)?

Ja ☐ nein ☐

Subjektive Verbesserung der Symptomatik im Vergleich zur Vorstellung bei 1. QST-Messung? (-10 bis + 10: 0 = keine Verbesserung, negative Werte = Verschlechterung, positive Werte = Verbesserung)

Beeinträchtigung im täglichen Leben durch CRPS? (0-10, 0 = gar nicht, 10 =vollständig abhängig)

Aktuelle Beschwerden Schmerz

Dauerschmerz	
ntermittierender Schmerz (z.B. nachts)	
Einschießende Schmerzattacken	
Orthostaseschmerz	
Nur Bewegungsschmerz (kein Spontanschmerz)	
Kein Schmerz	

Aktuelle Schmerzstärke (NAS 0-10):

Mittlere Schmerzstärke in den letzten 7 Tagen (NAS 0-10):

Aktuelle Beschwerden Sensorik

	Patient	Arzt
Schmerzen bei Kälteexposition („kalter Windhauch, Dusche etc.“)		
schmerzen bei Wärmeexposition („Sauna, Sommer Dusche etc.“)		
Kribbelparästhesien		
Taubheit		
Schmerzen bei leichter Berührung		
Verstärktes Schmerzempfinden auf schmerzhafte Reize		
Schmerz bei Berührung der kleinen Fingergelenke		
Keine		

Aktuelle Beschwerden Motorik

	Patient	Arzt
Reduzierte Muskelkraft der betroffenen Extremität		
Steifheit von Gelenken an der betroffenen Extremität		
Eingeschränkte Gebrauchsfähigkeit der betroffenen Extremität		
Greifen/ hantieren von Objekten nur unter Sicht möglich		
Muskelatrophie		
Unwillkürliches Zittern (Tremor)		
Unwillkürliche Fehlstellungen (Dystonie)		

Funktionseinschränkung der betroffenen Extremität

Der Patient soll 5 Aktivitäten nennen, die er vor Auftreten des CRPS regelmäßig durchgeführt hat, aber nun aufgrund der Schmerzen schwierig durchführen kann (siehe Moseley Graded motor imagery 2007). Dann sollen sie sagen, wie gut sie diese Aktivitäten nun durchführen können (auf einer Skala von 0-10, 0 = völlig unfähig, diese Aktivität durchzuführen, 10 = Aktivität kann ganz normal durchgeführt werden).

Aktivität	NRS

Mean NRS:

Range of motion Finger (nach Geertzen et al. 1994, Arch Phys Med Rehabil):

Finger-Hohlhand-Abstand: _________________ cm

Distanz Fingerspitze Dig 2 zur Handfläche: _________________ cm

Distanz Fingerspitze Dig 3 zur Handfläche: _________________ cm

Distanz Fingerspitze Dig 4 zur Handfläche: _________________ cm

Distanz Fingerspitze Dig 5 zur Handfläche: _________________ cm

Finger-Tapping (Bradykinese):

Betroffene Extremität(1. Messung) __________

Mean ____________

Betroffene Extremität (2. Messung) __________

Kontralaterale Extremität (1. Messung) __________

Mean ____________

Kontralaterale Extremität (2. Messung) __________

Durchführung des Quick-DASH

Sonstiges:

Schläfstörungen	
Arbeitsunfähig	
Sozialer Rückzug	

Aktuelle Beschwerden Autonom

	Patient	Arzt
Verändertes Hautkolorit		
Kältere Eytremität		
Wärmere Extremität		
Dauerhaftes Ödem		
Intermittierendes Ödem		
Verminderte Schweißsekretion		
Vermehrte Schweißsekretion		
Veränderte Behaarung		
Beschleunigtes Nagelwachstum		
Verlangsamtes Nagelwachstum		
Keine		

Hauttemperatur

**Betroffene Extremität:**	**Kontralaterale Extremität:**
**Dig I:**	**Dig I:**
**Dig II:**	**Dig II:**
**Dig III:**	**Dig III:**
**Dig IV:**	**Dig IV:**
**Dig V:**	**Dig V:**

Betroffene Extremität (MW): ☐

Kontralaterale Extremität (MW): ☐

Entzündungszeichen

Rötung	Patient	Arzt
Überwärmung		
Schwellung		
Schmerz		
Functio laesa		

Anzahl der vorliegenden Entzündungszeichen (Arzt) ☐

Aktuell dauerhafte Schmerzmedikation?

Ja ☐ nein ☐

Wenn ja, welche?

Trizyklika (Amitriptylin etc.)	
Ca^{2+}-Kanal modulierende Antikonvulsiva (Pregabalin, Gabapentin)	
Niederpotente Opioide	
Hochpotente Opioide	
Andere:	

Aktuell Begleittherapie?

Ja ☐ nein ☐

Wenn ja, welche?

Physiotherapie (inklusive Siegeltherapie, Lateralisation…)	
Ergotherapie	
Psychotherapie	
TENSS	
Interventionelle Therapie (Sympathikusblockaden, GLOAs)	
Invasive Therapie (SCS, DBS…)	

In der Vorgeschichte angewandte Therapie (Vgl. mit Daten aus 1. Messung!)

Trizyklika (Amitriptylin etc.)	
Ca-Kanal modulierende Antikonvulsiva (Pregabalin, Gabapentin)	
Niederpotente Opioide	
Hochpotente Opioide	
Physiotherapie (inklusive Siegeltherapie, Lateralisation…)	
Ergotherapie	
Psychotherapie	
TENSS	
Interventionelle Therapie (Sympathikusblockaden, GLOAs)	
Invasive Therapie (SCS, DBS…)	
Andere:	

Vorerkrankungen?

**Neu aufgetretene Komorbidität? Welche?**
**Sonstige neurologische Auffälligkeiten in der Untersuchung:**

Durchführung der QST
(vorher Hauttemperatur an der zu untersuchenden Lokalisation im Seitvergleich messen!)

Anlegen der Logger zur 24h-Temperaturmessung (mind. 8 h, möglichst tagsüber in 1-minütigen Abständen)!

Ggf. auch kürzeres Intervall möglich (8h).
