# Supplementary material for: Sensitization of the Nociceptive System in Complex Regional Pain Syndrome
Source: PLoS One. 2016 May 5;11(5):e0154553. doi: 10.1371/journal.pone.0154553 (PMC4858201; doi:10.1371/journal.pone.0154553)
Supplement: S2 Protocol — (PDF) [file pone.0154553.s002.pdf]

Sensitization of the nociceptive system in complex regional pain syndrome - study protocol (English)

All questions shoud be focused on CRPS / the affected extremity, if not otherwise specified!

Age (years):

Sex

male ☐ female ☐

Localization of CRPS:

Left: ☐ Right: ☐

Duration of CRPS (months):

Time between triggering event / beginning of pain and beginning of therapy

__________________ months

< 1 week ☐ < 1 month ☐ < 2 months ☐ > 2 months ☐

Duration between 1st QST-measurement and second measurement (months):

Triggering traumatic event

With or without preceding operation with ☐ without ☐

Nerve compression syndrome	
Fracture	
Joint injury	
Joint-/boneaffection	
Soft tissue syndrome	
Soft tissue injury (distortion etc.)	
spontaneous	

Positive scintigraphy (before 1st QST-measurement if applicable)?

yes ☐ no ☐

Subjective improvement of symptoms compared to initial visit at 1st QST-measurement? (-10 to + 10: 0 = no improvement, negative values = worsening, positive values = improvement)

Impairment in activities of daily living due to CRPS? (0-10, 0 = not at all, 10 = completely dependable)

Current complaints - pain

Continuous pain	
Intermittent pain (for example at night)	
Shooting pain	
Orthostatic pain	
Pain during movement without continuous pain	
No pain	

Current pain intensity (NAS 0-10):

Mean pain intensity in the last 7 days (NAS 0-10):

Current sensory complaints

	Patient	Doctor
Pain during cold exposure („cold wind puff, shower etc.“)		
Pain during warm exposure („sauna, summer, shower etc.“)		
Tingling paresthesias		
hypesthesia		
Pain during light touch		
Increased pain from painful stimuli		
Pain illicited by touch of finger joints		
None		

Current motor complaints

	Patient	Doctor
Reduced strength of the affected extremity		
Joint stiffness of the affected extremity		
Reduced usability of the affected extremity		
Grasping / handling of objects only possible if in sight		
Musle atrophy		
Involuntary shivering (tremor)		
Involuntary deformity (dystonia)		

Functional impairment of the affected extremity

The patient is supposed to name 5 activities, that he exercized regularly before the occurance of CRPS but that are more difficult now because of pain (see Moseley Graded motor imagery 2007). They are supposed to rate, how good they can perform this activity now (on an NRS 0-10, 0 = completely unable to perform the activity, 10 = activity can be performed as usual).

Activity	NRS

Mean NRS: ☐

Range of motion - Finger (according to Geertzen et al. 1994, Arch Phys Med Rehabil):

Finger-palm-distance: ________________ cm

Distance fingertip Dig 2 to the palm: ________________ cm

Distance fingertip Dig 3 to the palm: ________________ cm

Distance fingertip Dig 4 to the palm: ________________ cm

Distance fingertip Dig 5 to the palm: ________________ cm

Finger-Tapping (bradykinesia):

Affected extremity (1st measurement) __________

Mean ____________

Affected extremity (2nd measurement) __________

Contralateral extremity (1st measurement) __________

Mean ____________

Contralateral extremity (2nd measurement) __________

Completion of Quick-DASH

Miscellaneous:

Sleep disturbances	
Inability to work	
Social withdrawal	

Current complaints - autonomic

	Patient	Doctor
Change in skin color		
Colder extremity		
Warmer extremity		
Continuous edema		
Intermittent edema		
Reduced sweat excretion		
Increased sweat excretion		
Change in hairiness		
Accelerated nail growth		
Decelerated nail growth		
None		

Skin temperature

Affected extremity:	Contralateral extremity:
Dig I:	Dig I:
Dig II:	Dig II:
Dig III:	Dig III:
Dig IV:	Dig IV:
Dig V:	Dig V:

Affected extremity (mean): ☐

Contralateral extremity (mean): ☐

Inflammatory signs

Redness	Patient	Doctor
Heat		
Swelling		
Pain		
Functio laesa		

Amount of inflammatory signs present (Doctor) ☐

Currently continuous pain medication?

yes ☐ no ☐

if so, which one?

Tricyclic antidepressants (amitriptylin etc.)	
Ca^{2+} - channel modulationg anticonvulsants (pregabalin, gabapentin)	
Low potent opioids	
High potent opioids	
Other:	

Currently concomitant treatment?

yes ☐ no ☐

if so, which one?

Physiotherapy (including mirror therapy, lateralization…)	
Occupational Therapy	
Psychotherapy	
TENSS	
Interventional therapy (sympathetic blocks, GLOAs)	
Invasive treatment (SCS, DBS…)	

Treatment in past medical history (comparison with data from initial visit)

Tricyclic antidepressants (amitriptylin etc.)	
Ca^{2+} - channel modulationg anticonvulsants (pregabalin, gabapentin)	
Low potent opioids	
High potent opioids	
Physiotherapy (including mirror therapy, lateralization…)	
Occupational Therapy	
Psychotherapy	
TENSS	
Interventional therapy (sympathetic blocks, GLOAs)	
Invasive treatment (SCS, DBS…)	
Other:	

Past medical history?

**Newly occured comorbidity? Which one?**
**Other neurological abnormality in clinical testing:**

Completion of QST
(after examination of skin temperature of both sides at the proposed localization!)

Application of the loggers for the 24h-temperature measurement (at least 8 hours, during daytime in 1-minute intervals)!

Shorter interval possible, if applicable (8h).
